# Digital twin for Taekwondo athletes: integrating sports nutrition and psychological readiness using artificial intelligence

**DOI:** 10.3389/fpubh.2026.1822194

**Published:** 2026-04-22

**Authors:** Adam Tawfiq Amawi, Gerasimos V. Grivas, Walaa Jumah Alkasasbeh

**Affiliations:** 1 Department of Movement Sciences and Sports Training, School of Sports Science, The University of Jordan, Amman, Jordan; 2 Physical Education and Sports, Division of Humanities and Political Sciences, Hellenic Naval Academy, Piraeus, Greece; 3 Department of Physical Education, School of Sports Science, The University of Jordan, Amman, Jordan

**Keywords:** artificial intelligence, digital twin, psychological readiness, smart sport systems, sports nutrition, sustainable athlete performance, Taekwondo

## Abstract

**Background:**

Taekwondo is a high-intensity Olympic combat sport that requires the integration of physical performance, tactical decision-making, and psychological resilience. Athletes face unique challenges such as rapid weight management, fatigue accumulation, injury risk, and competitive anxiety. While sports nutrition and psychological readiness are critical determinants of performance, they are often addressed separately, creating a gap in holistic, individualized athlete monitoring systems.

**Methods:**

This narrative review synthesizes interdisciplinary evidence from sport science, nutrition, psychology, and artificial intelligence. A structured literature search was conducted across PubMed, Scopus, Web of Science, and Google Scholar, focusing on studies related to Taekwondo performance, weight-category nutrition strategies, psychological readiness, and AI-driven technologies including wearable systems, machine learning, and digital twin frameworks.

**Results:**

The findings indicate that AI-driven digital twin technology enables the integration of multidimensional athlete data, including nutritional intake, psychological state, training load, and physiological biomarkers (e.g., HRV and cortisol). These systems can generate actionable outputs such as readiness scoring, personalized nutrition strategies, early detection of fatigue and stress dysregulation, and prediction of injury or overtraining risk.

**Conclusion:**

Digital twin technology represents a promising framework for transforming Taekwondo athlete management from fragmented monitoring to a holistic, data-driven approach. Practically, this may support coaches in making real-time decisions regarding training load, weight management, recovery, and psychological interventions. However, further empirical validation, ethical considerations, and applied research are required to support real-world implementation in elite combat sport environments.

## 1 Introduction

Taekwondo is a high-intensity Olympic combat sport that demands exceptional physical fitness, rapid decision-making, and strong psychological resilience (1). Athletes are required to perform repeated explosive movements, maintain tactical awareness under pressure, and cope with competitive stressors (2) such as weight-category management (3), injury risk (4), and performance anxiety (5). Therefore, optimizing both physiological and psychological readiness is essential for maximizing competitive success in Taekwondo (6).

In recent years, sport science research has increasingly highlighted the importance of integrating sports nutrition and psychological preparation as complementary components of athlete performance (7). Adequate nutritional strategies support energy availability, recovery, body composition goals, and immune function (8), while psychological readiness influences motivation, focus, emotional regulation, and confidence during competition (9). Despite their interconnected roles, these domains are often addressed separately in practice, limiting the ability to develop holistic and individualized athlete support systems.

Recent developments in Taekwondo, including the introduction of electronic scoring systems and evolving competition rules, have further increased the complexity of performance analysis, requiring continuous updates in technical–tactical classification frameworks (10). This highlights the limitations of static and traditional approaches, which may struggle to keep pace with the rapidly changing and multidimensional nature of the sport. Similarly, traditional taekwondo training methods have been shown to rely heavily on subjective observation, limiting the provision of objective and real-time feedback for performance improvement (11). Recent AI-driven approaches integrating motion analysis and augmented reality have demonstrated the potential to deliver precise, real-time feedback, highlighting the limitations of conventional systems and reinforcing the need for advanced, data-driven monitoring frameworks (11). In this context, artificial intelligence may represent a critical turning point, enabling continuous, integrated, and predictive monitoring of athlete readiness. By moving from reactive to proactive decision-making, AI-driven systems such as digital twins have the potential to fundamentally transform how performance is assessed and optimized in Taekwondo.

At the same time, advances in digital health technologies and artificial intelligence (AI) have introduced new opportunities for personalized athlete monitoring and decision support (12, 13). One emerging concept is the Digital Twin, a virtual representation of a real-world athlete that continuously integrates physiological, behavioral, and psychological data to simulate performance states and predict future outcomes (14). Digital twin models have gained attention in healthcare and engineering (14), and their application in sport is rapidly expanding through the use of wearable sensors, biometric tracking, machine learning algorithms, and predictive analytics (15–17).

For Taekwondo athletes, the digital twin framework offers a promising approach to unify key performance determinants (18, 19), including nutritional status (19), training load (20), recovery indicators (21), and psychological readiness (1). AI-driven systems can potentially identify early signs of fatigue (22), stress dysregulation (23), or inadequate fueling (24), enabling coaches and practitioners to tailor interventions in real time. Furthermore, integrating psychological variables such as competitive anxiety, mental toughness, and self-regulation into digital twin models may enhance the precision of performance predictions and improve athlete wellbeing (25).

However, despite the growing interest in AI-based sport technologies, the application of digital twin approaches specifically within combat sports remains limited (26). There is a lack of synthesized evidence on how digital twins can effectively integrate sports nutrition and psychological readiness for Taekwondo athletes, and what challenges and future directions exist in this emerging field.

Therefore, the purpose of this narrative review is to explore the concept of digital twin technology in the context of Taekwondo performance, with a particular focus on the integration of sports nutrition and psychological readiness using artificial intelligence. This review aimed to summarize current developments, highlight practical applications, and propose future research pathways for building holistic, data-driven athlete support systems in combat sports.

## 2 Methods

### 2.1 Design

This study adopted a narrative review design to synthesize interdisciplinary evidence on Taekwondo performance science and the emerging application of artificial intelligence (AI)–driven digital twin technology. The review specifically focused on the integration of sports nutrition and psychological readiness as complementary components for athlete monitoring, performance optimization, and decision support within elite combat sport environments.

The narrative review approach was selected due to its flexibility in integrating diverse types of evidence and its suitability for exploring emerging and multidisciplinary topics, as recommended in previous methodological guidelines (27). In addition, to enhance the methodological rigor and transparency of the review process, the quality of the included studies and the overall structure of the review were guided using the Scale for the Assessment of Narrative Review Articles (SANRA), a validated tool designed to improve the quality and reporting of narrative review (28).

Research Framework (PEO)

To further enhance methodological clarity and conceptual organization, the research question of this narrative review was structured using the PEO framework as follows:

Population (P): Taekwondo athletes.

Exposure (E): Integration of sports nutrition, psychological readiness, and AI-driven digital twin technologies.

Outcome (O): Performance optimization, athlete monitoring, fatigue and injury risk prediction, and data-driven decision-making.

Based on this framework, the research question guiding this narrative review was: How can the integration of sports nutrition and psychological readiness through AI-driven digital twin technologies enhance performance optimization, athlete monitoring, and risk prediction in Taekwondo athletes?

### 2.2 Search strategy

A structured literature search was conducted across major scientific databases, including PubMed/MEDLINE, Scopus, Web of Science, and Google Scholar, to identify peer-reviewed research relevant to Taekwondo and combat sport performance.

The search covered studies published between 2000 and January 2026, and the final search was conducted in January 2026.

The search targeted studies addressing the physiological and competitive demands of Taekwondo, nutrition and weight-management challenges in weight-category sports (such as low energy availability, RED-S, hydration strategies, and supplement use), psychological readiness and stress regulation factors, as well as technological innovations involving AI, wearable sensing, machine learning, and digital twin frameworks in sport and health contexts.

Search terms were combined using Boolean operators and included variations of “taekwondo” or “combat sport” alongside keywords related to nutrition, psychological readiness, mental fatigue, anxiety, self-regulation, artificial intelligence, wearable technology, and digital twin modeling. A summary of the search strategy is presented in Table 1.

**Table 1:** Summary of the literature search strategy and database queries.

Database	Search terms (example)	Filters applied
PubMed/MEDLINE	(“taekwondo” OR “combat sport”) AND (nutrition OR “energy availability” OR RED-S OR hydration OR supplements) AND (“psychological readiness” OR anxiety OR “mental fatigue” OR self-regulation) AND (“artificial intelligence” OR “wearable technology” OR “digital twin”)	English, peer-reviewed
Scopus	(“taekwondo” OR “combat sport”) AND (nutrition OR psychology) AND (“AI” OR “machine learning” OR wearable OR “digital twin”)	Articles, reviews
Web of Science	(“taekwondo”) AND (performance OR fatigue OR anxiety) AND (“artificial intelligence” OR “machine learning”)	English
Google Scholar	“taekwondo performance” AND nutrition AND psychology AND (“digital twin” OR AI)	Broad search

### 2.3 Eligibility criteria

Studies were considered eligible for inclusion if they examined Taekwondo or comparable weight-category combat sports such as judo or mixed martial arts, with clear relevance to Taekwondo-specific performance demands. Eligible research included investigations focusing on nutrition-related variables such as energy availability, rapid weight loss, hydration status, and supplementation, as well as psychological determinants including competitive anxiety, mood state, attention, mental fatigue, and mental toughness. In addition, studies presenting evidence on AI-enabled monitoring systems, predictive analytics, wearable technologies, or digital twin concepts applicable to individualized athlete modeling were included. Studies were excluded if they were not related to Taekwondo or comparable combat sports, did not address nutrition, psychological, or AI-related variables relevant to performance, were non-peer-reviewed opinion or editorial articles, or lacked sufficient methodological detail to support interpretation.

Non-peer-reviewed opinion pieces and sources lacking sufficient methodological detail were generally excluded, although foundational conceptual papers and high-quality narrative or systematic reviews were retained to support the development of the proposed framework.

### 2.4 Study selection and synthesis approach

**Figure 1:**
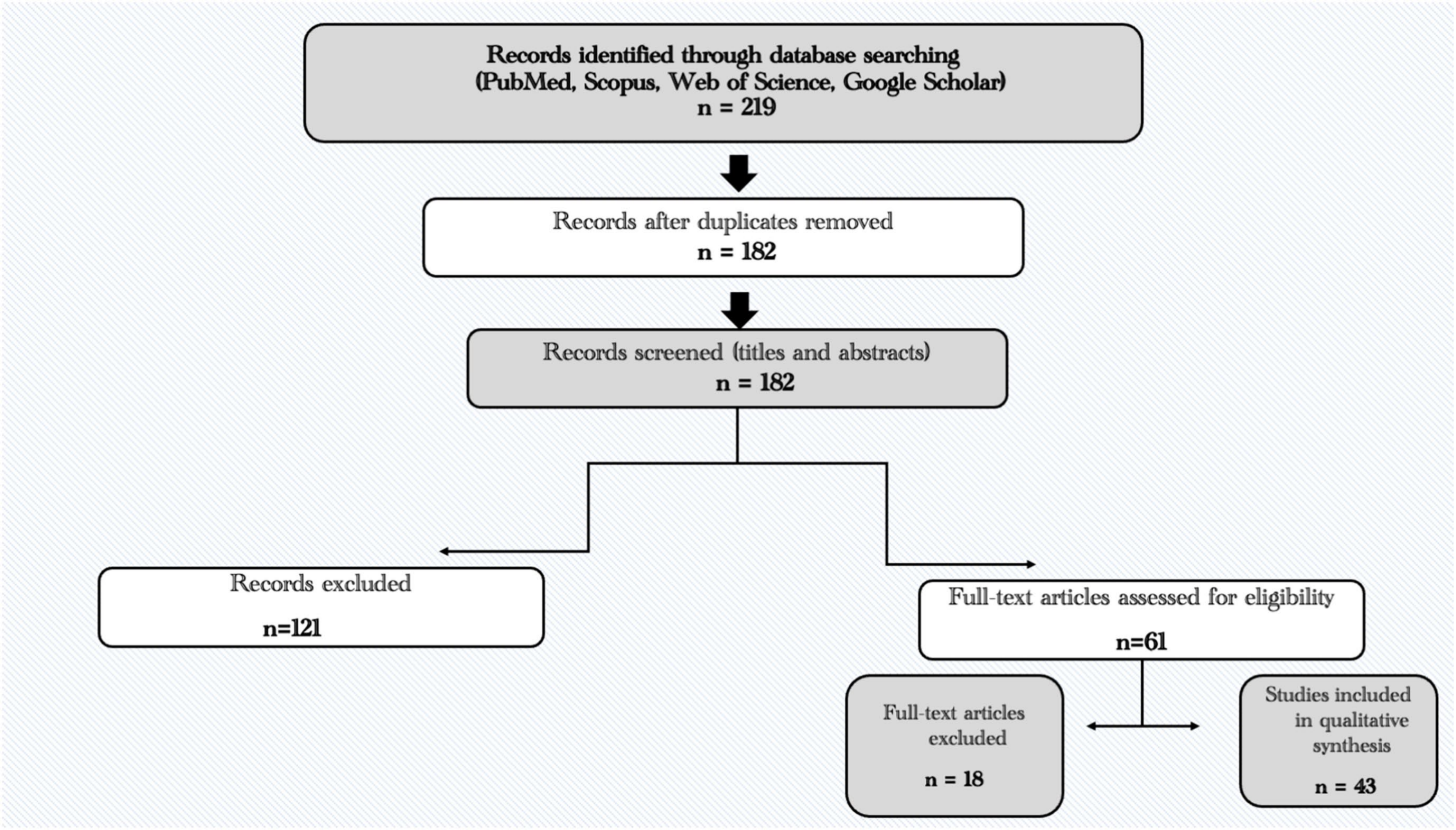
Simplified PRISMA flow diagram of study selection.

Retrieved records were initially screened based on titles and abstracts, followed by full-text evaluation to confirm relevance to the aims of the review. Two reviewers independently screened the titles and abstracts of the retrieved studies, followed by full-text assessment. Any disagreements were resolved through discussion and consensus. Evidence was synthesized qualitatively through a thematic approach and organized into four integrated domains: the physiological and competitive demands of Taekwondo, sport-specific nutritional strategies and weight-category constraints, psychological readiness and stress-related determinants of performance, and the role of AI-driven applications leading toward a Taekwondo-specific digital twin framework linking multidimensional inputs, AI processing layers, and actionable outputs. The study selection process is illustrated in Figure 1.

### 2.5 Quality assessment of included studies

To enhance methodological rigor and transparency, the quality of the included studies was assessed using key principles derived from the Scale for the Assessment of Narrative Review Articles (SANRA). The evaluation focused on clarity of objectives, literature coverage, scientific reasoning, and data presentation. Given the narrative nature of this review, a structured qualitative assessment was conducted rather than a formal risk-of-bias analysis. Due to the narrative nature of the review and the heterogeneity of included studies, a category-based qualitative assessment was deemed more appropriate than individual risk-of-bias scoring. A summary of the methodological quality assessment of the included studies is presented in Table 2.

**Table 2:** Summary of methodological quality of included studies (based on SANRA principles).

Study type	Clarity of objective	Literature coverage	Scientific reasoning	Data presentation	Overall quality
Narrative reviews	Moderate–High	High	Moderate	Moderate	Good
Experimental studies	High	Moderate–High	High	High	Good
Observational studies	Moderate	Moderate	Moderate	Moderate	Acceptable
AI and digital twin studies	Moderate	Moderate–High	Moderate	Moderate	Acceptable–Good

## 3 Results

The results are presented in a structured format using concise bullet points to enhance clarity and readability, while interpretations and interactions among findings are addressed in the Discussion section.

### 3.1 Physiological and competitive demands of taekwondo

#### 3.1.1 Energy system requirements

Taekwondo performance relies on a combination of anaerobic and aerobic energy pathways (29). During matches, athletes execute rapid and explosive movements, particularly repeated high-intensity kicking techniques and short attack–defense exchanges (30).

These actions require immediate energy production, primarily supported by anaerobic metabolism, including:

- ATP–PC system
- Anaerobic glycolysis (31, 32)

At the same time, the aerobic system plays a critical role in recovery between high-intensity bursts and across successive rounds (33, 34). Aerobic fitness contributes to faster phosphocreatine resynthesis, improved fatigue resistance, and enhanced overall match endurance (35). Therefore, elite Taekwondo athletes must develop the capacity to repeatedly generate powerful anaerobic bursts while maintaining efficient aerobic recovery (31).

The modern competitive style of Taekwondo, with frequent kicking sequences and continuous movement patterns, places significant physiological stress on athletes (36). This highlights the importance of precise monitoring of energy expenditure, fatigue accumulation, and nutritional strategies within an integrated digital framework (18).

#### 3.1.2 Weight-category challenges

A defining feature of Taekwondo, as in many combat sports, is competition within strict weight categories (37). As a result, athletes often engage in rapid weight loss practices prior to competition in an attempt to gain a perceived advantage by competing in a lower weight class (38).

Although rapid weight reduction may offer short-term competitive benefits, it is associated with substantial risks for both performance and health (39). Rapid weight loss can lead to:

- Decreased energy availability
- Dehydration
- Electrolyte imbalance
- Impaired muscular strength
- Reduced cognitive functioning (39)

In addition, these practices may increase the likelihood of injury, illness, and psychological distress (40).

Consequently, evidence-based nutritional planning and safe weight management strategies are essential components of Taekwondo athlete preparation. Digital twin models supported by artificial intelligence may provide valuable tools for tracking body mass fluctuations, recovery status, and metabolic indicators, thereby reducing the negative consequences of unsafe weight-cutting behaviors.

#### 3.1.3 Match stress and decision-making demands

Beyond physical demands, Taekwondo imposes considerable psychological and cognitive stress during competition (6, 41). Matches require athletes to process rapidly changing tactical situations, anticipate opponents’ actions, and make split-second decisions while under physiological fatigue (6).

As fatigue increases throughout the bout, attentional focus and decision-making accuracy may decline (42), potentially compromising technical execution and tactical performance. Furthermore, competitive stressors can negatively affect performance, including:

- Anxiety
- Pressure to win
- Fear of making mistakes (43)

Therefore, cognitive readiness and psychological preparedness are critical determinants of success in Taekwondo (6). Integrating psychological variables such as competitive anxiety (44), mental toughness (45), and self-regulation (46) into athlete monitoring systems may enhance performance prediction and support targeted interventions (47). In this context, digital twin approaches offer promising opportunities to incorporate both physiological and psychological indicators into a holistic model of athlete readiness (48).

### 3.2 Sports nutrition strategies in Taekwondo

Sports nutrition plays a central role in supporting the physiological demands of Taekwondo (49), a combat sport characterized by:

- Repeated high-intensity actions
- Rapid recovery requirements
- Strict weight-category constraints (50)

Optimal nutritional strategies are essential not only for enhancing performance but also for maintaining athlete health, reducing fatigue, and supporting psychological readiness (2, 8, 30, 31). In the context of emerging digital twin applications, nutrition-related data may represent a key component for individualized monitoring and decision-making (51).

#### 3.2.1 Energy availability and performance

Adequate energy availability is fundamental for sustaining training adaptation (29), competition performance, and overall wellbeing in Taekwondo athletes (52, 53). Energy availability refers to the amount of dietary energy remaining for physiological functions after accounting for exercise energy expenditure (54).

Combat sport athletes are particularly vulnerable to low energy availability (LEA) due to high training loads combined with intentional weight-control practices (55). Persistent LEA increases the risk of Relative Energy Deficiency in Sport (RED-S) (56), a syndrome associated with:

- Impaired metabolic function
- Hormonal disturbances
- Reduced bone health
- Decreased immune function
- Compromised performance capacity (57)

In Taekwondo, insufficient fueling may lead to:

- Reduced power output
- Slower recovery
- Impaired concentration
- Increased injury risk (58)

Therefore, maintaining adequate energy intake across training and competition phases is critical. Monitoring energy availability through AI-supported systems could provide early detection of RED-S risk and support individualized nutritional interventions (59).

#### 3.2.2 Carbohydrate periodization for combat performance

Carbohydrates represent a primary fuel source for high-intensity intermittent sports such as Taekwondo (60). Given the repeated anaerobic bursts required during matches, adequate muscle glycogen stores are essential for sustaining explosive kicking performance and tactical movement patterns (35, 61).

Carbohydrate periodization, which involves adjusting carbohydrate intake according to training intensity and competition demands, has gained attention as a practical strategy for combat sport athletes (62, 63). During heavy training and pre-competition phases, higher carbohydrate availability supports maximal performance capacity and reduces fatigue (64).

Pre-competition fueling strategies typically emphasize:

- Carbohydrate-rich meals
- Optimization of glycogen storage
- Adequate energy availability during bouts (65)

Post-match and post-training recovery nutrition plays a key role in:

- Glycogen replenishment
- Muscle repair
- Training adaptation (66, 67)

Within a digital twin framework, carbohydrate intake patterns could be integrated with training load and fatigue markers to optimize fueling recommendations in real time (68).

#### 3.2.3 Hydration and thermoregulation

Hydration status is another critical factor influencing Taekwondo performance, particularly given the common practice of rapid weight loss through dehydration (69). Even mild dehydration can impair physiological and cognitive function, negatively affecting endurance, strength, and thermoregulation (70).

In combat sports, dehydration has been shown to:

- Reduce reaction time
- Increase perceived exertion
- Compromise decision-making accuracy (71, 72)

Furthermore, impaired thermoregulation increases the risk of heat-related stress during intense bouts and training sessions (73). Therefore, structured hydration strategies before, during, and after competition are essential for maintaining performance and safety (74). Digital monitoring tools, including wearable sensors, may provide valuable hydration-related indicators that can be incorporated into athlete digital twin models (75).

#### 3.2.4 Supplements in taekwondo athletes

Dietary supplements are widely used among combat sport athletes to enhance performance, support recovery, or manage fatigue (76, 77). However, supplement use in Taekwondo must be approached cautiously, balancing evidence-based benefits with potential health risks and anti-doping considerations.

Among the most commonly discussed ergogenic aids are:

- Caffeine—may improve alertness, reaction speed, and high-intensity performance (78, 79)
- Creatine—supports short-duration explosive power and repeated sprint ability (80–82)
- Beta-alanine—enhances buffering capacity and reduces fatigue during repeated high-intensity efforts (83–85)

While these supplements have demonstrated efficacy in certain athletic contexts, their application in Taekwondo should be individualized based on training phase, athlete tolerance, and competition demands.

Importantly, athletes face a significant doping risk due to contamination or misuse of supplements (86). Therefore, supplement strategies must rely on high-quality evidence, professional supervision, and certified products. In future digital twin systems, supplement intake could be integrated with physiological responses and readiness indicators (68, 87), supporting safer and more personalized performance optimization.

Collectively, these nutrition strategies highlight the importance of individualized fueling, hydration, and supplementation in Taekwondo. Integrating such variables into AI-driven digital twin models may enhance performance prediction, reduce health risks, and support holistic athlete readiness.

### 3.3 Psychological readiness in Taekwondo athletes

Psychological readiness is a critical determinant of success in Taekwondo (88, 89), a sport characterized by rapid exchanges, high-pressure decision-making, and intense emotional demands (90). While physical conditioning and nutrition provide the physiological foundation for performance (91), psychological factors often differentiate winners from losers in elite-level combat sports (92).

Competitive outcomes in Taekwondo are strongly influenced by an athlete’s ability to:

- Regulate anxiety
- Maintain focus
- Manage emotions
- Sustain cognitive performance under fatigue (93–95)

Given the increasing interest in holistic athlete monitoring, psychological readiness represents a key domain that should be integrated into AI-driven digital twin models alongside physiological and nutritional variables.

#### 3.3.1 Competitive anxiety and stress regulation

Competitive anxiety is one of the most prevalent psychological challenges in Taekwondo athletes, particularly in the pre-fight period (41). Athletes frequently experience heightened arousal, worry, and physiological stress responses before competition, which may influence performance execution (96).

While moderate arousal can enhance alertness and readiness (97), excessive anxiety may impair reaction time, disrupt coordination, and reduce tactical effectiveness (98). Stress regulation is therefore essential for optimal performance (99), requiring athletes to achieve an appropriate balance between activation and control (100, 101).

Moreover, combat sports often elicit strong physiological stress markers, including:

- Elevated cortisol
- Autonomic nervous system responses (102–104)

Monitoring stress regulation through biomarkers and psychological assessments may provide valuable indicators of readiness within digital twin frameworks (105, 106).

#### 3.3.2 Emotion regulation and mental toughness

Taekwondo competition involves intense emotional fluctuations, including:

- Aggression
- Fear of injury
- Frustration
- Pressure to win (41)

Effective emotion regulation allows athletes to maintain composure (107), prevent impulsive errors (108), and execute tactical strategies under stress (109).

Psychological factors such as mood patterns and emotion regulation play a critical role in performance, as both emotional stability and regulation skills have been identified as key determinants of performance readiness and execution (110, 111).

Mental toughness is widely recognized as a key psychological trait in combat sports (112), reflecting the capacity to persist through discomfort (113), maintain confidence (114), and remain resilient in challenging situations (115). Athletes with higher mental toughness are better equipped to cope with setbacks (116), sustain motivation, and perform consistently in high-stakes bouts (116).

Intrinsic motivation has been identified as a key determinant of sustained athletic performance, as it promotes long-term engagement, continuous improvement, and psychological resilience, whereas extrinsic motivation tends to produce short-term performance gains with less enduring impact (117). Integrating emotion-related variables into digital athlete models may improve understanding of performance variability and support targeted psychological interventions (118).

#### 3.3.3 Attention, focus, and decision-making

Elite Taekwondo performance requires continuous attentional control and rapid decision-making (119). Athletes must anticipate opponents’ actions, adapt tactics instantly, and execute precise motor responses within fractions of a second (120).

Cognitive performance under pressure is particularly important in Taekwondo due to the speed and unpredictability of combat interactions (42). Stress, fatigue, and distractions can reduce attentional focus (121), leading to delayed reactions and tactical misjudgments. Therefore, attention regulation and cognitive readiness are essential psychological components that should be considered alongside physiological metrics in AI-based monitoring systems.

#### 3.3.4 Mental fatigue and performance decline

Mental fatigue has emerged as an important factor influencing athletic performance, particularly in sports requiring high cognitive engagement (122, 123).

In Taekwondo, sustained mental fatigue during training and competition may:

- Impair alertness
- Slow reaction time
- Increase tactical errors (124).

Mental fatigue may also interact with physical fatigue (125), amplifying perceived exertion and reducing the athlete’s ability to maintain high-intensity performance across rounds (123). As a result, psychological exhaustion can compromise both technical execution and decision-making accuracy (122).

Digital twin systems that incorporate indicators of mental fatigue could provide early warnings of performance decline and support individualized recovery strategies (126).

#### 3.3.5 Psychological skills training interventions

Psychological skills training (PST) interventions are widely used to enhance mental readiness and performance in combat sport athletes (127). These strategies aim to strengthen coping mechanisms, improve focus, and optimize emotional regulation before and during competition (44, 128).

Common evidence-based psychological interventions include:

- Imagery training—enhances confidence and tactical preparation through mental rehearsal (129)
- Self-talk techniques—reinforce motivation, focus, and emotional control (130)
- Mindfulness-based approaches—improve attentional stability and reduce competitive anxiety (131)
- Breathing and relaxation strategies—support arousal regulation and stress recovery (132)

Such interventions have shown promising effects on competitive anxiety reduction, improved concentration, and enhanced resilience (129–132). Incorporating PST outcomes into digital twin frameworks may allow for more personalized mental training recommendations based on real-time readiness indicators.

Overall, psychological readiness in Taekwondo encompasses anxiety regulation, emotional control, cognitive focus, and resistance to mental fatigue. Integrating these psychological dimensions with physiological and nutritional data through artificial intelligence–based digital twin models may provide a more comprehensive understanding of athlete performance and enable optimized individualized interventions. A summary of these psychological and nutritional challenges is presented in Table 3.

**Table 3:** Psychological and nutritional challenges in weight-category combat sports.

Challenge	Impact on athletes	Digital twin monitoring opportunity
Rapid weight loss practices	Mood disturbance, dehydration, reduced cognition	Early warning system for unsafe weight cuts
Low energy availability (RED-S)	Hormonal disruption, fatigue, injury risk	Personalized energy availability prediction
Competitive anxiety	Impaired decision-making, performance decline	AI-triggered psychological intervention
Mental fatigue	Slower reaction speed, tactical errors	Cognitive readiness scoring via biomarkers

### 3.4 The nutrition–psychology interaction in combat sports

In combat sports such as Taekwondo, nutritional status and psychological readiness are deeply interconnected (133). Unlike many other athletic disciplines, weight-category sports impose unique physiological and mental stressors that can amplify the interaction between fueling strategies, mood regulation, and cognitive performance (93, 133, 134).

Rapid weight loss practices, commonly used to meet weight-class requirements, have been associated with significant psychological consequences, including mood disturbance (135), irritability (136), heightened stress (137), and impaired concentration (138). Athletes undergoing aggressive weight-cutting may experience reduced emotional stability (135), which can negatively influence tactical decision-making during competition.

Similarly, low carbohydrate availability often resulting from restrictive dieting may increase perceived exertion, reduce motivation, and contribute to cognitive decline under fatigue (139, 140). Lifestyle-related factors such as sleep quality further influence this interaction, as evidence suggests that poor sleep is associated with impaired emotional regulation and less favorable nutritional attitudes, highlighting the complex interplay between sleep, nutrition, and psychological functioning (141).

Since Taekwondo requires fast reactions and attentional control, inadequate fueling can compromise both physical output and mental sharpness (69, 133).

The use of ergogenic aids such as caffeine further illustrates the nutrition–psychology overlap (142, 143). While caffeine may enhance alertness and reaction speed, excessive intake can exacerbate anxiety and arousal dysregulation (144, 145), particularly in athletes already vulnerable to pre-competition stress.

Moreover, the pressure of maintaining a competitive weight may increase the risk of disordered eating behaviors and long-term psychological burden (146). Therefore, integrated approaches that simultaneously address nutritional adequacy and mental wellbeing are essential for sustainable performance optimization in Taekwondo.

This further emphasizes the need for integrated approaches, as empirical evidence has demonstrated a significant relationship between dietary habits and the psychological state of athletes, as well as a positive association between sports nutrition knowledge and healthy dietary behaviors state (138, 147).

### 3.5 Artificial intelligence applications in Taekwondo

Artificial intelligence has rapidly emerged as a transformative tool in sport science (148), offering advanced methods for athlete monitoring, performance prediction, and individualized decision support (13). In Taekwondo, AI applications are increasingly relevant due to the sport’s dynamic physiological demands, tactical complexity, and psychological intensity (149).

Digital health technologies can improve athletes’ nutrition knowledge and health behaviors, but their limited impact on psychological readiness highlights the need for integrated AI-driven systems such as digital twin frameworks (150).

#### 3.5.1 Wearable technology and athlete monitoring

Wearable devices provide continuous access to physiological and behavioral indicators such as heart rate (HR), heart rate variability (HRV), sleep quality, and training load metrics (151, 152). These data streams allow practitioners to monitor recovery status, stress regulation, and readiness fluctuations across training cycles.

In Taekwondo, wearable-based monitoring can support early detection of fatigue accumulation, autonomic imbalance, and insufficient recovery factors closely linked to injury risk and performance decline (13, 153). HRV, particularly RMSSD, is recognized as a reliable non-invasive biomarker of autonomic regulation, stress, and recovery in athletes, supporting its use in routine monitoring and training adaptation strategies (154).

#### 3.5.2 Computer vision and match-performance analytics

Computer vision systems have expanded opportunities for objective match-performance assessment (155). Through video-based AI analysis, key competitive variables such as kicking speed, movement patterns, reaction timing, and tactical behaviors can be quantified with high precision (156–158). These technologies enable coaches to evaluate performance beyond subjective observation, supporting data-driven tactical preparation and individualized technical feedback. For instance, AI-driven video analysis and motion-tracking systems have been shown to enhance the objective assessment of technical and tactical actions in combat sports by quantifying performance metrics and reducing reliance on subjective evaluation methods (159).

#### 3.5.3 Machine learning models for prediction

Machine learning approaches are increasingly used to predict complex performance outcomes in sport (160). In Taekwondo, predictive models may assist in forecasting injury risk (161), estimating fatigue states (162), and generating readiness profiles based on multidimensional athlete data (163). Such models can integrate physiological, nutritional, and psychological indicators to provide individualized risk alerts and optimize training interventions (164, 165). For example, machine learning models have been successfully applied to predict injury risk based on workload and physiological variables such as heart rate and training load metrics, demonstrating their ability to identify key risk factors and support injury prevention strategies in athletes (166).

#### 3.5.4 Natural language processing (NLP) for mental-state monitoring

Natural language processing offers innovative possibilities for monitoring athlete psychological wellbeing (167). Athlete diaries, self-reported stress logs, and qualitative feedback can be analyzed using sentiment detection and linguistic pattern recognition (168).

NLP-based systems may identify early signals of burnout, anxiety, or emotional distress, complementing physiological monitoring and enhancing holistic athlete care (169). For example, digital mental health platforms supported by artificial intelligence and machine learning have been used to assess and monitor psychological states in athletes through continuous behavioral and self-reported data, enabling early detection of psychological strain (170).

However, while such digital tools provide valuable insights into athletes’ psychological states, they are most effective when integrated with conventional rehabilitation approaches rather than used alone (171). Technology-based interventions further demonstrate this limitation, as mobile-assisted applications have been shown to significantly reduce fear-related responses, despite having no significant effect on intrinsic motivation (172).

Similarly, a mobile learning application was shown to significantly improve nutrition knowledge, although it did not produce significant changes in intrinsic motivation (173). These findings highlight that although digital tools can enhance specific cognitive or emotional aspects, they may be insufficient to influence overall psychological readiness without integration into comprehensive monitoring systems.

This reinforces the need for integrated AI-driven frameworks, such as digital twin systems, that combine psychological, physiological, and behavioral data to optimize athlete wellbeing and performance.

### 3.6 Digital twin framework for Taekwondo athletes

The concept of the digital twin represents one of the most promising innovations in sport performance science (174, 175). A digital twin is defined as a dynamic virtual model of an athlete that is continuously updated through real-time data inputs, enabling simulation, prediction, and individualized decision support (14, 174, 176). In sport contexts, such systems enable the integration of multidimensional data streams and support predictive modeling and continuous model refinement, allowing adaptive decision-making in athlete monitoring (17).

Building on this concept, the proposed system represents an advanced AI-driven monitoring and decision-support platform, which can be considered an evolutionary step toward a fully operational digital twin in Taekwondo.

In Taekwondo, digital twin technology offers a unique opportunity to integrate key determinants of performance nutrition (177), psychology (178), and physical readiness within a single AI-driven framework (17), within a unified, data-driven framework that supports holistic athlete monitoring and optimization.

#### 3.6.1 What is a digital twin in sport?

A sport digital twin is not merely a performance dashboard, but rather an adaptive computational representation of the athlete (174). By combining wearable sensor data (179), training metrics (180), nutritional patterns (181), and psychological indicators (182), the digital twin can model the athlete’s current state and predict future outcomes. Accordingly, this approach enables a shift from reactive to proactive performance management. In advanced implementations, digital twins can simulate potential responses to training loads (183), nutritional strategies (177), or recovery interventions (184), allowing practitioners to test different scenarios before applying them in practice. Furthermore, the model is continuously refined through iterative data integration, thereby improving its predictive accuracy over time.

Importantly, in practical implementations, this process follows a structured data pipeline in which raw data are collected from wearable sensors (185), including heart rate monitors (186), GPS systems (187), and biochemical markers (188), preprocessed and filtered to remove noise, transformed into meaningful features, and subsequently analyzed using machine learning algorithms to generate predictions and decision-support outputs (189–191).

#### 3.6.2 Core components of a Taekwondo digital twin

A Taekwondo-specific digital twin framework may be conceptualized as a multi-layered system that integrates physiological, psychological, and performance-related data to support individualized athlete monitoring and optimization (20, 153, 192). This framework typically consists of three interconnected layers: inputs, AI-driven processing, and outputs. Although primarily conceptual, this structure closely reflects practical data-driven architectures used in intelligent athlete monitoring systems, encompassing real-time data acquisition, processing pipelines, and adaptive feedback generation.

In real-world sport environments, such architectures are implemented using integrated platforms combining wearable technologies (185, 193), including Polar (194), WHOOP (194), Catapult (195), video analysis systems (196), and cloud-based data processing pipelines that enable continuous data streaming and analysis (197).

##### 3.6.2.1 Inputs

The input layer incorporates multidimensional indicators that reflect the athlete’s current condition (198), including nutrition variables such as energy intake, hydration status, supplement use, and weight fluctuations relevant to weight-category demands (8). In addition, it integrates key psychological indicators, including competitive anxiety levels (199, 200), mood state (200), sleep quality (201), and mental fatigue (202). This layer also captures sport-specific performance metrics, such as training load (203), match statistics (204), and physiological biomarkers of recovery (205), including heart rate variability (HRV), cortisol responses, and recovery indices (206).

These data are typically acquired through multimodal sources, including wearable sensors, mobile applications (207), self-reported questionnaires (208), and video-tracking systems (209), which require synchronization and standardization to ensure temporal alignment across data streams.

##### 3.6.2.2 AI processing layer

The AI processing layer applies advanced computational techniques to interpret and integrate the complex data streams collected from the input layer (210). This includes multimodal data fusion to combine nutritional, psychological, and performance-related information into a unified athlete profile (211), as well as pattern recognition and anomaly detection to identify early signs of maladaptation, fatigue accumulation, or performance decline (212–214). Furthermore, personalized prediction algorithms are employed to generate individualized forecasts tailored to the athlete’s unique characteristics (215), thereby supporting real-time monitoring and evidence-informed decision-making.

In addition, adaptive learning mechanisms can be incorporated to enable continuous model refinement based on incoming data streams and athlete responses (216). Through feedback loops, the system dynamically updates its predictions and recommendations, enhancing accuracy over time and allowing for context-aware adjustments (216). This adaptive capability ensures that the AI processing layer not only interprets current states but also evolves alongside the athlete’s physiological and psychological changes, thereby improving long-term performance optimization and injury prevention (217).

From a technical perspective, this layer may utilize machine learning approaches (218), including supervised learning through regression models and decision trees, deep learning architectures based on neural networks, and time-series analysis to model athlete readiness and predict injury or fatigue risk (191, 219, 220). However, integrating heterogeneous data sources presents challenges such as missing data, sensor noise, data heterogeneity, and the need for robust data fusion algorithms capable of handling asynchronous inputs (221–225).

##### 3.6.2.3 Outputs

The output layer translates AI-driven insights into actionable recommendations that can support coaches, practitioners, and athletes in optimizing training and recovery strategies (211). These outputs may include readiness scoring systems to guide performance-related decisions (226), personalized nutrition recommendations aligned with the athlete’s physiological demands (227), and automated psychological intervention triggers such as alerts for stress overload or burnout risk (167, 228). In addition, the system may provide predictive forecasting of injury susceptibility (229), overtraining, and burnout (168), enabling timely preventive adjustments and sustainable performance management. Taken together, these outputs reflect an intelligent, data-driven decision-support ecosystem. In applied settings, these outputs are typically delivered through user-friendly dashboards or mobile applications (18, 230), enabling coaches to make real-time adjustments to training load (231), recovery strategies (232), and nutritional interventions (173). Despite these advances, practical implementation remains challenging due to issues related to data privacy, system integration, and the need for validation in real-world elite sport environments.

While the proposed framework incorporates key elements of digital twin architecture, its current design remains primarily oriented toward advanced monitoring and decision support. This positioning represents a practical and scalable step toward the future realization of fully integrated digital twin systems in combat sports. An overview of the digital twin components and their applications is presented in Table 4.

**Table 4:** Key components of a taekwondo digital twin framework.

Domain	Key variables (examples)	Digital twin application	Expected output
Sports Nutrition	Energy intake, hydration status, supplement use, weight fluctuations	Continuous nutrition monitoring	Personalized fueling and weight-management strategies
Psychological Readiness	Competitive anxiety, mood state, mental fatigue, sleep quality	Real-time psychological profiling	Stress alerts and tailored mental interventions
Performance Metrics	Training load, match statistics, tactical decision speed	AI-based performance modeling	Readiness scoring and performance prediction
Physiological Biomarkers	HRV, cortisol, recovery indices	Biomarker-driven fatigue detection	Overtraining and injury risk forecasting
AI Processing Layer	Data fusion, anomaly detection, predictive algorithms	Individualized athlete digital twin simulation	Decision support for coaches and practitioners

**Figure 2:**
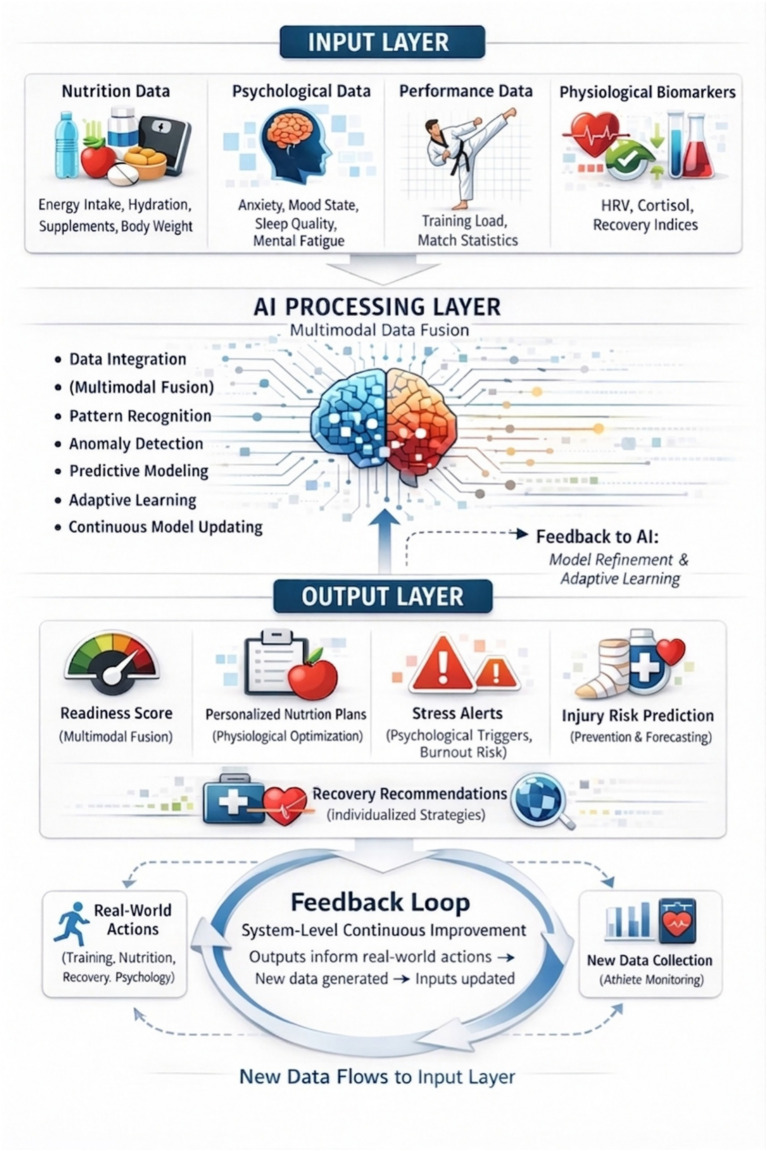
Taekwondo digital twin framework.

Ultimately, the integration of AI-driven digital twins in Taekwondo has the potential to enhance performance sustainability, athlete wellbeing, and long-term competitive success. The digital twin paradigm represents a next-generation framework for combat sport preparation, offering a holistic integration of nutrition, psychology, and performance analytics. Future research should prioritize empirical validation, ethical implementation, and real-world feasibility to ensure that such systems can be effectively translated into elite Taekwondo environments. As shown in Figure 2, the proposed digital twin framework integrates multidimensional input data, AI-driven processing, and actionable outputs within a continuous feedback loop.

**Figure 3:**
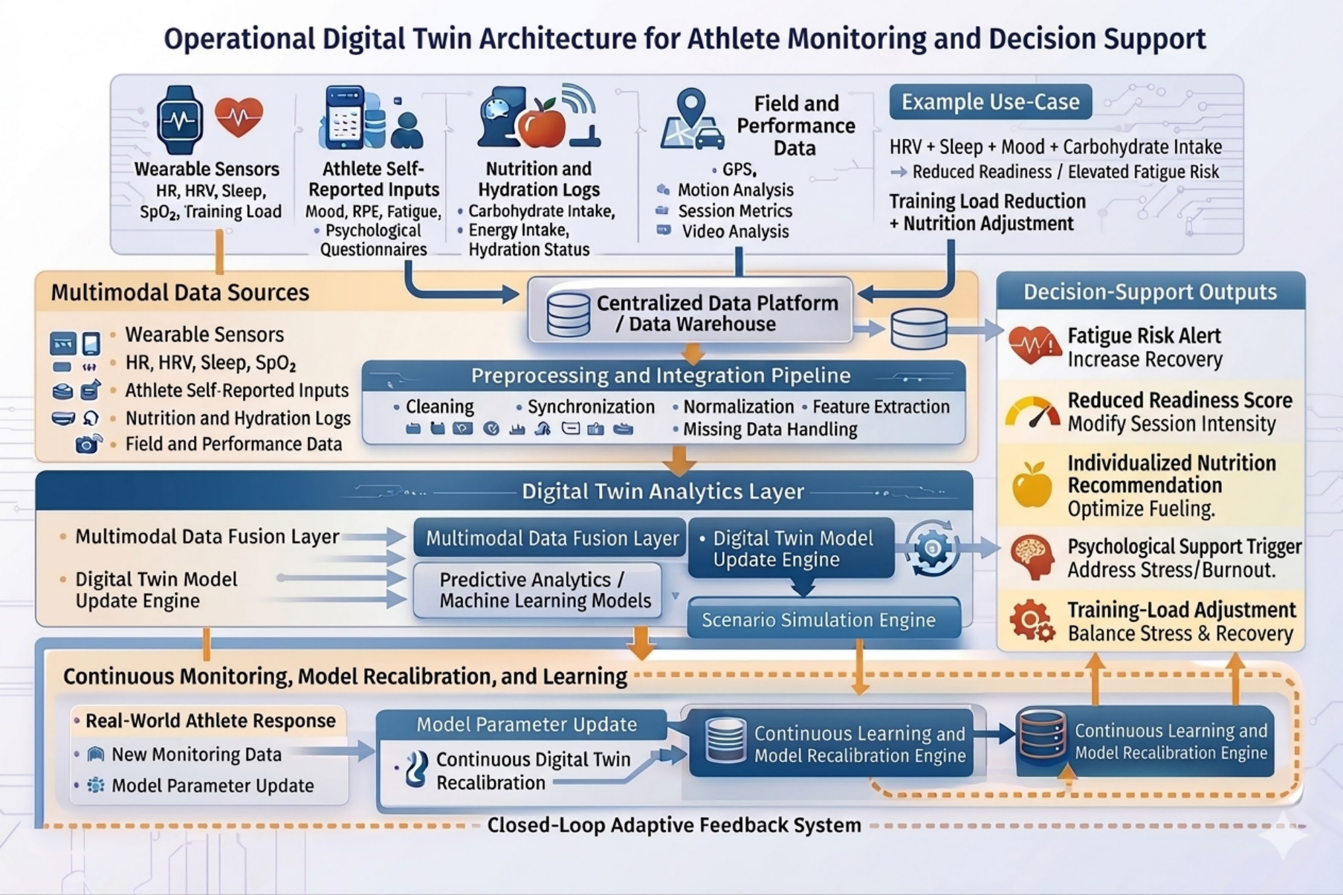
Operational digital twin architecture for athlete monitoring and decision support in Taekwondo.

To further clarify the practical implementation of the proposed framework, a detailed operational digital twin architecture is presented in Figure 3. The diagram illustrates the complete data pipeline, including multimodal data acquisition from wearable sensors, self-reported inputs, nutritional logs, and performance data; preprocessing and integration steps such as data cleaning, synchronization, normalization, and feature extraction; and a centralized data platform enabling multimodal data fusion. The digital twin analytics layer incorporates machine learning models, continuous model updating, and scenario simulation to generate actionable outputs such as fatigue risk alerts, readiness scores, and individualized intervention strategies. A closed-loop feedback mechanism supports continuous monitoring and model recalibration based on real-world athlete responses.

#### 3.6.3 Operational perspective of the digital twin framework

To provide a more concrete representation of the proposed system, a typical operational workflow can be described within a Taekwondo training context. During a competition preparation week, physiological data such as HR and HRV are continuously collected via wearable devices, including systems such as Polar and WHOOP, while sleep quality and training load are automatically tracked. In parallel, athletes record daily nutritional intake and complete brief psychological assessments assessing anxiety, mood state, and mental fatigue.

These heterogeneous data streams are first synchronized within a centralized platform to ensure temporal alignment, as physiological data are collected continuously whereas psychological and nutritional inputs are recorded at discrete time points. Preprocessing procedures, including noise filtering and missing data imputation, are applied to improve data quality. The system then performs feature extraction, including indicators such as RMSSD for HRV, cumulative training load, and estimated energy availability, followed by multimodal data fusion to generate an integrated athlete profile.

Machine learning models subsequently analyze the combined dataset to identify patterns associated with fatigue, under-recovery, or suboptimal fueling. For example, a simultaneous decrease in HRV, elevated perceived fatigue, and reduced carbohydrate intake may trigger a fatigue-risk alert. Based on this output, the system provides actionable recommendations, such as reducing training intensity, increasing carbohydrate availability, or implementing psychological recovery strategies. Importantly, the system operates as a continuous feedback loop, where new incoming data refine the model and update predictions over time, reflecting the adaptive nature of a true digital twin.

## 4 Discussion

This narrative review highlights the growing potential of artificial intelligence (AI)–driven digital twin technology as an innovative paradigm for enhancing performance optimization and athlete wellbeing in Taekwondo. As an Olympic combat sport, Taekwondo requires a complex interaction of explosive physical capacity, high-intensity intermittent effort, rapid tactical decision-making, and psychological resilience under competitive pressure (30, 32). Previous evidence has demonstrated that Taekwondo athletes face sport-specific stressors such as accumulated fatigue, elevated injury risk, and the physiological constraints associated with weight-category competition, reinforcing the need for continuous monitoring of readiness and recovery (29, 58).

From a nutritional perspective, weight-management practices represent a critical challenge in Taekwondo and comparable combat sports. Rapid weight loss strategies and low energy availability are widely documented as risk factors for impaired physiological function, hormonal disruption, and increased susceptibility to injury (38, 39, 56). Hydration status is another key determinant, as dehydration has been linked to reductions in cognitive performance, judgment, and sport-specific decision-making factors essential in elite Taekwondo competition (69, 73, 74). In addition, dietary supplement use is prevalent in combat sports, yet requires evidence-based oversight due to potential health concerns and inadvertent doping risks associated with contaminated products (76, 77, 86). These findings emphasize the importance of integrating individualized nutrition monitoring into future athlete-support systems.

Psychological readiness represents an equally essential dimension of performance in combat sports. Competitive anxiety, mood disturbances, and mental fatigue have been shown to negatively influence attentional control, reaction speed, and tactical execution during high-pressure competitive settings (96, 112, 122). At the same time, interventions such as psychological skills training, mindfulness-based strategies, and emotional regulation approaches have demonstrated positive effects on coping capacity and performance-related outcomes in athletes (44, 107, 131). However, despite growing evidence, psychological indicators remain insufficiently integrated into real-time monitoring frameworks, highlighting the need for more comprehensive athlete-centered models.

In this context, digital twin technology offers a promising solution by enabling the continuous integration of multidimensional athlete data, including nutrition variables, psychological markers, physiological biomarkers, and training-load metrics, into a dynamic individualized digital representation (14, 17, 174). Advances in wearable technologies further support the feasibility of capturing real-time indicators such as heart rate variability (HRV) and cortisol responses, which are strongly associated with stress, recovery, and competitive readiness (104, 206). Machine learning methods and multimodal data fusion can facilitate pattern recognition, anomaly detection, and predictive modeling to forecast injury risk, burnout, or overtraining before clinical symptoms emerge (204, 212, 229).

Despite these opportunities, digital twin applications in Taekwondo remain in an early developmental stage. Most existing work is conceptual or derived from broader sport and health contexts, with limited longitudinal and experimental research validating digital twin systems within real-world elite combat sport environments (174, 175). Furthermore, the implementation of AI-based athlete digital twins raises ethical concerns related to privacy, informed consent, data governance, algorithmic bias, and transparency of decision-support outputs, which must be addressed prior to widespread adoption (118, 233).

Overall, this review suggests that AI-driven digital twins may represent a next-generation framework capable of redefining elite Taekwondo preparation through the integration of nutrition optimization, psychological readiness monitoring, and physiological recovery assessment. Future progress will require interdisciplinary collaboration among sport scientists, nutritionists, sport psychologists, data engineers, and coaching practitioners to ensure that these systems are valid, interpretable, ethically governed, and practically applicable within athlete support infrastructures.

## 5 Practical applications for coaches and practitioners

From an applied perspective, AI-driven digital twin systems hold substantial potential to support elite coaching teams and sport science practitioners in Taekwondo (149, 153, 234). These systems may enable comprehensive competition-week readiness monitoring through the integration of multidimensional physiological and psychological indicators, providing a more accurate representation of the athlete’s current condition. In addition, digital twins can contribute to safer weight-management practices by guiding nutritional regulation and minimizing the risks associated with rapid weight loss and relative energy deficiency in sport (RED-S) (177, 235, 236), which remain critical concerns in weight-category combat sports. Furthermore, real-time psychological readiness feedback may assist practitioners in optimizing anxiety regulation (237), attentional focus (238), and emotional control during high-pressure competitive contexts (239). Personalized recovery strategies can also be enhanced through continuous fatigue and sleep analytics (240, 241), allowing for timely adjustments in training load and regeneration protocols. Importantly, the incorporation of decision-support functions within digital twin platforms may facilitate data-driven coaching interventions (233), thereby improving strategic planning and athlete management in high-performance environments.

## 6 Future directions and research gaps

Despite the growing interest in artificial intelligence–driven digital twin technology within sport science, its application in Taekwondo and other weight-category combat sports remains in an early developmental stage. Current literature is largely conceptual, with limited empirical studies validating digital twin frameworks in real-world elite training and competition settings. One major research gap involves the lack of longitudinal and experimental investigations examining how continuous integration of nutritional, psychological, physiological, and performance data can enhance readiness optimization and decision-making over time.

Future research should prioritize the development of Taekwondo-specific digital twin models that account for the unique physiological demands, rapid weight-management practices, and high cognitive–emotional stress experienced by athletes. In particular, more evidence is needed on the feasibility of combining wearable-derived biomarkers (such as, HRV, cortisol, hydration indicators) with psychological monitoring tools to generate actionable and individualized intervention strategies. Additionally, interdisciplinary collaboration among sport nutritionists, sport psychologists, data scientists, and coaches will be essential to translate these systems into applied athlete-support environments.

Ethical and practical challenges also represent critical gaps in the current knowledge base. Issues related to data privacy, athlete autonomy, algorithmic bias, and the interpretability of AI-based predictions must be addressed before widespread adoption is possible. Finally, future studies should explore how digital twins can be implemented within sustainable, cost-effective infrastructures that are accessible beyond highly resourced elite sport organizations. Overall, advancing digital twin applications in Taekwondo will require robust validation, ethical governance, and applied research that bridges technological innovation with athlete health and performance outcomes.

## 7 Conclusion

The integration of artificial intelligence–driven digital twin technology into Taekwondo represents a transformative advancement in combat sport performance science. By combining multidimensional data streams related to sports nutrition, psychological readiness, training load, recovery biomarkers, and competitive stress responses, digital twin frameworks offer a holistic and individualized approach to athlete monitoring and decision support. Such systems have the potential to enhance readiness optimization, improve safe weight-management strategies, support targeted psychological interventions, and reduce the risk of injury, burnout, and overtraining.

Despite the promising theoretical and applied benefits, the implementation of digital twins in elite combat sport environments remains at an early stage, with limited empirical validation and practical deployment. Future research should focus on developing sport-specific models, conducting longitudinal experimental studies, and addressing ethical challenges related to data privacy, athlete autonomy, and real-world feasibility. Ultimately, AI-enabled digital twins may serve as a next-generation paradigm for sustainable performance enhancement and athlete wellbeing in Taekwondo and other weight-category combat sports.

## 8 Limitations

Despite the promising conceptual value of AI-driven digital twin frameworks in Taekwondo, several limitations should be acknowledged. First, this work is based on a narrative review approach rather than a systematic review or meta-analysis, which may limit the comprehensiveness of evidence coverage and does not allow for quantitative synthesis of outcomes. Second, empirical research validating digital twin applications specifically within elite Taekwondo environments remains scarce, with most available studies being theoretical or derived from broader combat sport or health-related contexts. Additionally, the heterogeneity of methodologies, wearable technologies, biomarker assessments, and psychological measurement tools across studies makes it difficult to establish standardized protocols for implementation. Therefore, conclusions should be interpreted as a conceptual foundation that requires further experimental validation and sport-specific refinement.

## 9 Ethical considerations and data governance

The integration of digital twin systems in elite combat sports also raises important ethical and practical considerations. Digital twins rely on continuous collection of sensitive physiological, psychological, and behavioral data, which introduces challenges related to athlete privacy, informed consent, and secure data governance. Moreover, AI-based predictive algorithms may be subject to bias, limited interpretability, and uncertainty, potentially influencing coaching or medical decisions if not applied responsibly. Ensuring athlete autonomy, transparency in algorithmic decision-making, and adherence to ethical standards in data management will be essential before widespread adoption of digital twin technologies in Taekwondo. Future research should therefore incorporate ethical frameworks and multidisciplinary oversight to ensure that digital twin systems enhance athlete wellbeing without compromising rights, trust, or safety.
